# Artificial Intelligence-Assisted Score Analysis for Predicting the Expression of the Immunotherapy Biomarker PD-L1 in Lung Cancer

**DOI:** 10.3389/fimmu.2022.893198

**Published:** 2022-07-01

**Authors:** Guoping Cheng, Fuchuang Zhang, Yishi Xing, Xingyi Hu, He Zhang, Shiting Chen, Mengdao Li, Chaolong Peng, Guangtai Ding, Dadong Zhang, Peilin Chen, Qingxin Xia, Meijuan Wu

**Affiliations:** ^1^ Department of Pathology, The Cancer Hospital of the University of Chinese Academy of Sciences (Zhejiang Cancer Hospital), Hangzhou, China; ^2^ Institute of Basic Medicine and Cancer, Chinese Academy of Sciences, Hangzhou, China; ^3^ 3D Medicines Inc., Shanghai, China; ^4^ The Second Clinical Medical College, Zhejiang Chinese Medical University, Hangzhou, China; ^5^ Department of Pathology, Affiliated Cancer Hospital of Zhengzhou University, Zhengzhou, China; ^6^ School of Computer Engineering and Science, Shanghai University, Shanghai, China

**Keywords:** PD-L1, NSCLC, automated scoring, AI, pathological diagnosis

## Abstract

Programmed cell death ligand 1 (PD-L1) is a critical biomarker for predicting the response to immunotherapy. However, traditional quantitative evaluation of PD-L1 expression using immunohistochemistry staining remains challenging for pathologists. Here we developed a deep learning (DL)-based artificial intelligence (AI) model to automatically analyze the immunohistochemical expression of PD-L1 in lung cancer patients. A total of 1,288 patients with lung cancer were included in the study. The diagnostic ability of three different AI models (M1, M2, and M3) was assessed in both PD-L1 (22C3) and PD-L1 (SP263) assays. M2 and M3 showed improved performance in the evaluation of PD-L1 expression in the PD-L1 (22C3) assay, especially at 1% cutoff. Highly accurate performance in the PD-L1 (SP263) was also achieved, with accuracy and specificity of 96.4 and 96.8% in both M2 and M3, respectively. Moreover, the diagnostic results of these three AI-assisted models were highly consistent with those from the pathologist. Similar performances of M1, M2, and M3 in the 22C3 dataset were also obtained in lung adenocarcinoma and lung squamous cell carcinoma in both sampling methods. In conclusion, these results suggest that AI-assisted diagnostic models in PD-L1 expression are a promising tool for improving the efficiency of clinical pathologists.

## Introduction

Immunotherapy is one of the important pharmacological options for lung cancer treatment (1, 2). As a major immune checkpoint biomarker, programmed cell death ligand 1 (PD-L1) expression is widely considered a gold standard for predicting the response to immunotherapy (3). In the clinical context, the choice of immunotherapeutic strategies mainly depends on the levels of PD-L1 expression in tumor cells. Normally, a higher expression level of PD-L1 in tumor cells is associated with the patient’s better response to immunotherapy (4). Thus, efficient and accurate assessment of PD-L1 expression plays a critical role in cancer immunotherapy. However, there are still some challenges for the traditional methods of interpretation of PD-L1-positive tumor cells.

Moreover, the tumor microenvironment (TME) is a dynamic structure that is considered to play a role in tumor initiation and progression (5, 6). The extensive interaction among the TME, tumor cells, and immune cells provides novel opportunities for therapeutic strategies of cancer (7). Previous studies have shown multiple biomarkers in the TME and their predictive role in disease outcomes (6). Moreover, biomarkers within the TME may help us identify the beneficiaries of immunotherapy (6). Thus, the characteristics of the TME and its components make them ideal candidates for cancer-specific pathological diagnosis and precision treatments.

The application space of digital pathology and scanners has expanded greatly with the development of artificial intelligence (AI). AI-based automatic learning and diagnosis models could easily solve complex problems during medical image analysis (8). Deep learning (DL) and machine learning could promote further optimization of the AI-based image-processing models (9). DL methods have shown great advantages in image identification and classification (10), especially in cell classification (11), cancer detection (12), pathological diagnosis (13), and characterization of the spatial organization of immune cells in the TME (14). Previous studies have shown the application of DL in the analysis of multiple biomarkers in immunohistochemistry (IHC) staining, including epidermal growth factor receptor, human epidermal growth factor receptor 2, and Ki67 (15–17). AI-based quantitative diagnosis could also reduce the disadvantages of traditional methods, such as time consumption, lack of reproducibility, and interobserver variability (18, 19). Thus, AI-based automatic diagnosis models for tumor-specific biomarkers have promising application prospects in precise stratified medicine.

Specifically, several studies have shown the evaluation of AI models for PD-L1 expression in non-small cell lung carcinoma (20, 21). Whole-slide images of PD-L1-stained slides were automatically annotated with an AI model (22). PD-L1 expression on tumor cells and immune cells was further labeled, detected, and calculated. Interestingly, the algorithms of image-based scoring are highly consistent with those of pathologists when assessing PD-L1 expression (23). However, existing models have poor specificity and accuracy for pathological sections with low PD-L1 expression, especially at tumor proportion score (TPS) cutoff values of 1% (24, 25).

In this study, we explored and optimized three different AI model-based workflows for automatically detecting the positive PD-L1 expression in both 22C3 and SP263 assays. A highly accurate performance of the AI-assisted DL diagnostic models was shown in lung adenocarcinoma and lung squamous cell carcinoma of both sampling methods, especially for PD-L1 expression at 1% cutoff. Moreover, the M2 workflow was able to further improve the accuracy of the results. Our results indicate that AI-based diagnostic models are a promising approach to assist pathologists in the accurate diagnosis of PD-L1 expression.

## Materials and Methods

### Materials

A total of 1,288 formalin-fixed, paraffin-embedded lung cancer samples from Zhejiang Cancer Hospital were obtained. All samples were processed in the 3DMed Clinical Laboratory (accredited by CAP and CLIA). Among these, 1,204 samples were prepared and stained using the PD-L1 IHC 22C3 pharmDx assay (Dako, Carpenteria, CA, USA) developed on the Dako Autostainer Link 48 platform according to the kit’s manufacturer recommendations. In total, 84 samples were prepared and stained using PD-L1 IHC SP263 assays (Ventana Medical Systems, Tucson, AZ, USA) developed on the Ventana BenchMark platform.

Clinicopathological characteristics (*e*.*g*., age, gender, and tumor type) of lung cancer were included in this study. The detailed patient demographics and PD-L1 results are summarized in **Table 1**. This study was approved by the Zhejiang Cancer Hospital Ethics Committee (IRB-2020-310 and IRB-2021-439). All slides were digitized by a KFBIO FK-Pro-120 slide scanner at ×20 magnification (0.475 μm/pixel). Furthermore, 627 PD-L1 (22C3)-staining whole-slide images (WSIs) were used to develop prediction models, and the remaining WSIs were used as test sets (**Table 1**). The training WSIs were manually annotated by two graduate students majoring in pathology, and all annotations were confirmed by pathologists. The TPSs of all slides were estimated by one trained pathologist and confirmed by another.

**Table 1:** Clinicopathological characteristics of lung cancer.

Characteristic	Training set(*N* = 627)	Validation set (22C3)(*N* = 577)	Validation set (SP263)(*N* = 84)
**Age, years**			
Average	61	62	59
Range	25–91	21–91	30–83
**Gender**			
Male	385	308	43
Female	242	269	41
**Tissue source**			
Lung	538	532	79
Lymph nodes	53	22	4
Other	36	23	1
**Sampling methods**			
Surgical operation	305	282	30
Needle biopsy	220	198	48
Other biopsies	74	63	4
Pleural effusion	12	18	1
Other	16	16	1
**Tumor tissue type**			
Lung adenocarcinoma	425	497	67
Lung squamous cell carcinoma	102	67	16
Other	100	13	1
**TPS**			
<1%	316	442	63
1–50%	167	88	10
≥50%	144	47	11
**CPS**			
<1%	145	278	38
≥1%	471	280	45
NA	11	19	1

TPS, tumor proportion score; CPS, combined positive score; NA, not available.

### TPS Algorithm

TPS was calculated as the percentage of viable tumor cells exhibiting weak to strong partial or complete membranous staining. In order to accurately calculate TPS, we proposed a two-stage workflow based on DL: first, classification models were used to detect patches containing tumor cells, and then an object detection model was used to locate and count the tumor cells.

### The Development of Classification Models

**Figure 1:**
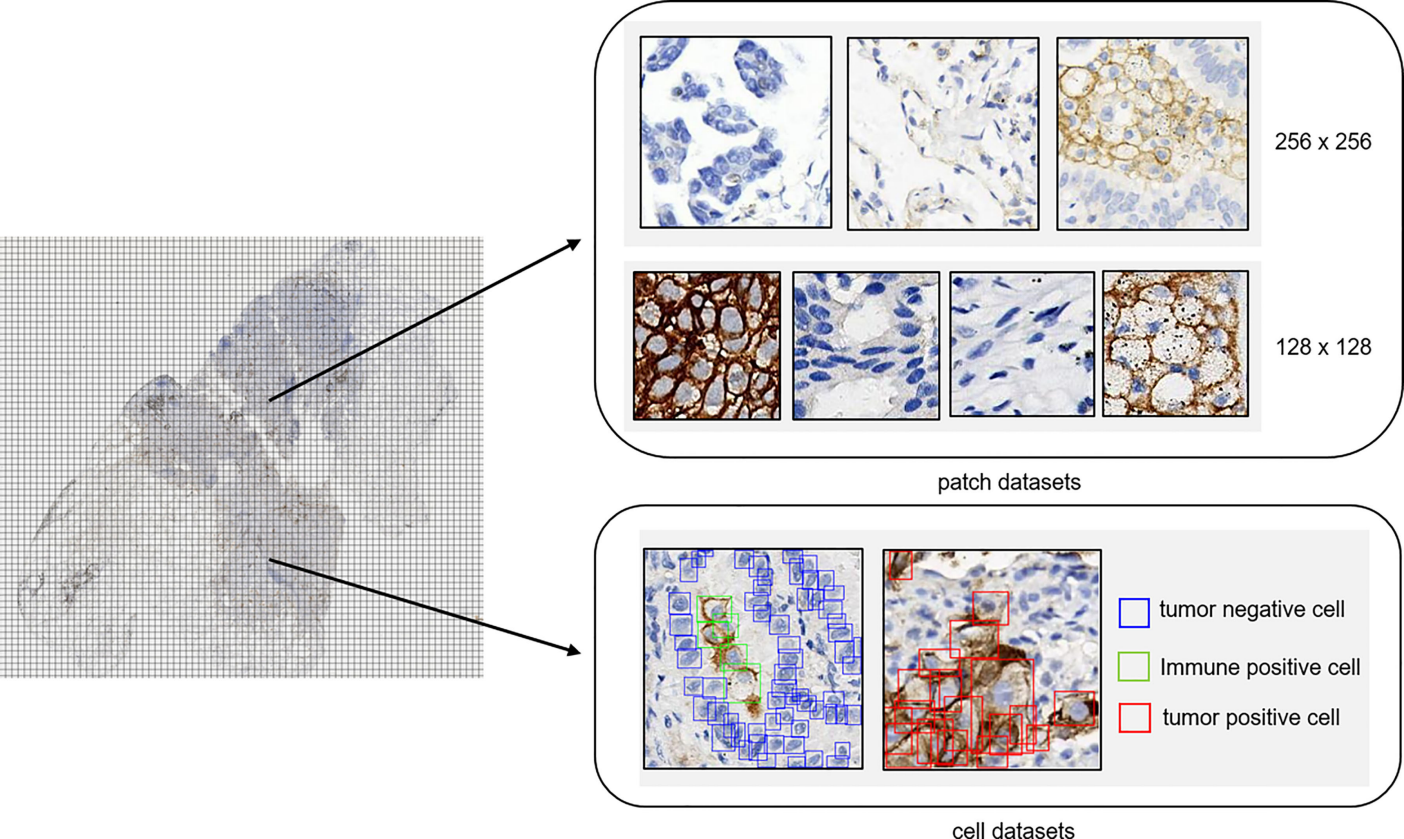
Annotation dataset for tumor detection. Patch datasets and cell datasets annotated in the whole-slide images of PD-L1 staining. Both 256 × 256 patch size and 128 × 128 patch size were included in the patch datasets. In the cell datasets, PD-L1-positive tumor cells, PD-L1-positive immune cells, and PD-L1-negative tumor cells were labeled with different colors.

Among the samples, more than 600 slides were selected for patch classification. To distinguish tumor cells containing patches from the others, we proposed two different classification models using convolutional neural networks with different input image sizes, namely, 256 × 256 pixels and 128 × 128 pixels, respectively (**Figure 1**). To train the networks with an input image size of 256 × 256 pixels, patches were randomly obtained and checked from the WSI annotation areas and then grouped into three categories: patches containing tumor cells but no PD-L1 positive immune cells (category 1: 124,459), patches containing both PD-L1 negative tumor cells and PD-L1 positive immune cells (category 2: 14,069; immune cells include macrophages and lymphocytes), and patches excluding tumor cells [category 3: 131,672; this category was comprised of various no-tumor tissues, including negative immune cells (macrophages and lymphocytes), hemorrhage, necrosis tissue, and stromal cells] (**Supplementary Figure S1**). Two classification models were constructed for different tasks: model (12_3), trained with patches among all three categories, was designed to classify the patches into tumor cell-containing patches (category 1 + category 2) and no-tumor cell-containing patches (category 3) and model (1_2), using category 1 and category 2 as training data sets, was constructed to classify the tumor cell-containing patches into PD-L1-positive immune cell-containing patches or no-PD-L1-positive immune cell-containing patches.

As for the development of networks with an input image size of 128 × 128, patches with a size of 128 × 128 were randomly obtained and checked from the WSI annotation areas and then grouped into four categories: patches containing PD-L1-positive tumor cells (category 4: 37,583), patches containing PD-L1-negative tumor cells (category 5: 45,107), patches containing PD-L1-positive immune cells (category 6: 38,192; immune cells including macrophages and lymphocytes), and other patches [category 7: 65,786; this category was comprised of various no-tumor tissues including negative immune cells (macrophages and lymphocytes), hemorrhage, necrosis tissue, and stromal cells]. The patches were fed into the network for model training.

All datasets for model training were randomly split into training and validation sets in a ratio of 8:2. Data augmentation was performed during the training by random flip, rotation, and blur. We employed MobileNetV2 architecture pretrained on ImageNet as the basic classification model. The MobileNetV2 architecture was the same as in a previous paper (26), with the depth multiplier and width multiplier both being 1 and using global max pooling for feature extraction. We removed the top layer (classify layer) in the original model and added a dropout layer and a dense layer for our task.

### Cell Detection

We built our own object detection model based on the YOLO head to quantitatively classify, locate, and count the PD-L1 tumor cells. We used CSPDarknet53 as our backbone (27); our feature network resembled BiFPN (28). Cell tags were labeled into patches of 128 × 128 pixel size and were grouped into PD-L1-negative tumor cells (105,508), PD-L1-positive tumor cells (24,523), and PD-L1-positive immune cells (10,429) (**Supplementary Figure S1**). In the data augmentation step, the same strategies were applied. In the training step, we used fivefold cross-validation and label smoothing (0.1) to avoid overfitting. From the predicted output of the cell detection model, only PD-L1-positive tumor cells and PD-L1-negative cells remained to calculate the TPS.

### Flow Chart of the Study

**Figure 2:**
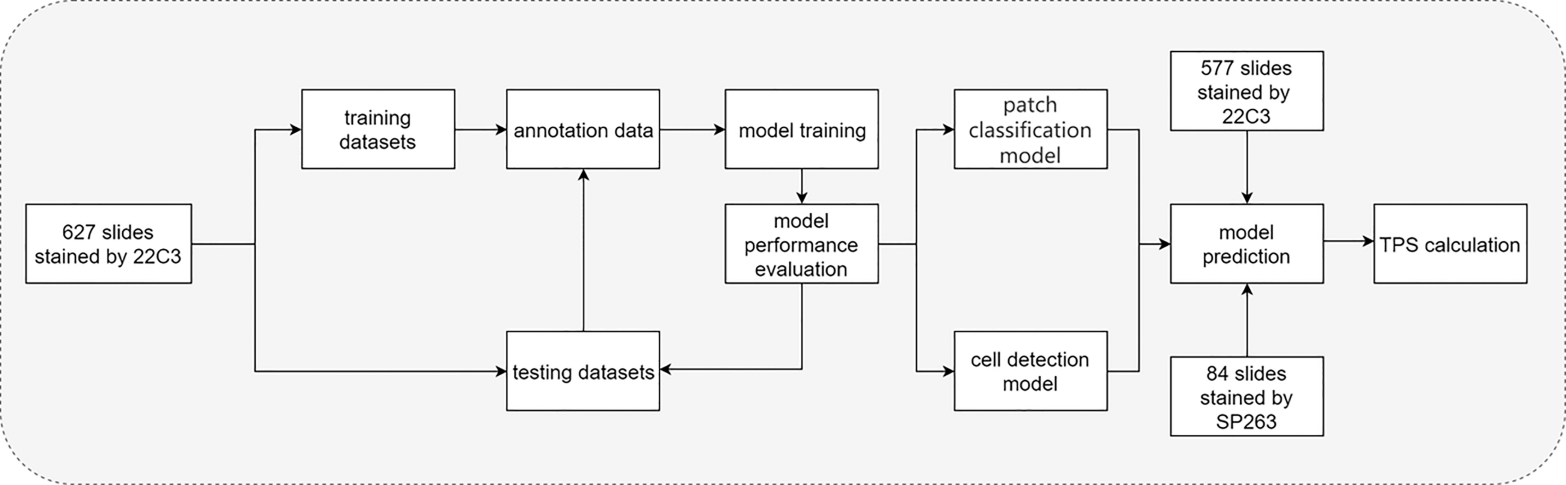
Flow chart of the study. Flow chart of tumor proportion score assessed with artificial intelligence-based diagnostic models in the pathological sections of lung cancer tissue samples stained with PD-L1 (22C3).

The flow chart of the DL model is shown in **Figure 2**. In brief, 627 pathological sections of lung cancer tissue staining samples with PD-L1 (22C3) were used for the DL model building. During the training, a subset of the WSIs was first selected, annotated, and fed into the network for training. Then, the remaining WSIs were used for the evaluation and refinement of the DL model performance. Both the established classification model and the cell detection model were then combined for the next test. Then, 577 slides stained by PD-L1 (22C3) and 84 slides stained by PD-L1 (SP263) were used for the independent testing of the DL model. Based on the cell detection of the combined DL models, the TPS of WSIs was obtained.

### WSI Inference Workflow

**Figure 3:**
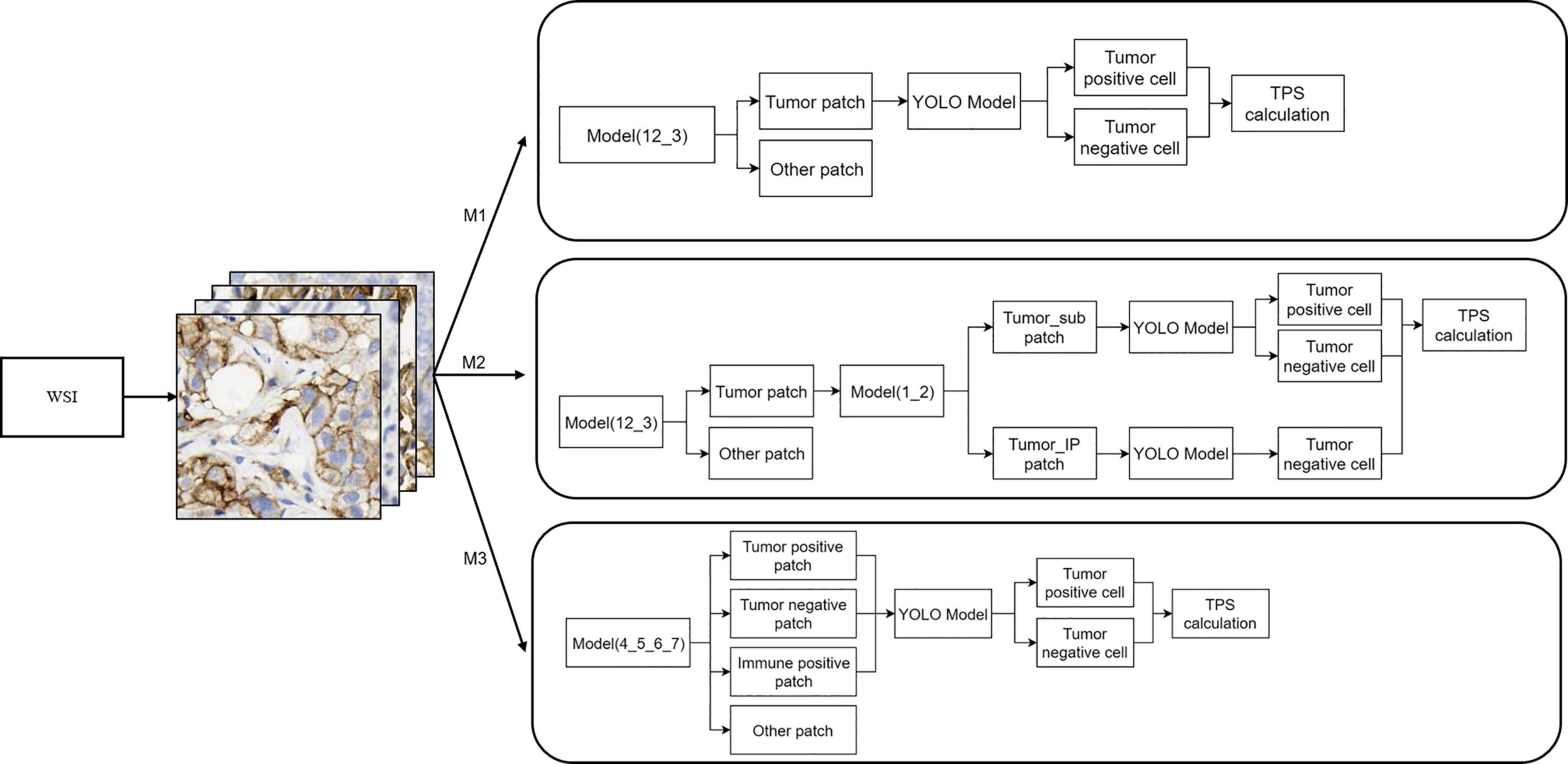
Whole-slide image inference workflow. A whole-slide image was analyzed with different artificial intelligence model-based workflows (M1, M2, and M3). The detailed information of these three workflows is shown here.

The WSI inference workflow is shown in **Figure 3**. Three different workflows (top, M1; middle, M2; and bottom, M3) were used for the calculation of the TPS. For M1, the patches were divided into tumor and other regions by model (12_3); the YOLO model was further used for the detection of PD-L1-positive and PD-L1-negative tumor cells. M2 included further algorithm optimization based on M1. The tumor patches in M2 workflow were further divided into patches with or without PD-L1-positive immune cells by model (1_2), and then the YOLO model was used for the detection of PD-L1-positive or PD-L1-negative tumor cells inside the patches. Compared with M1, M2 could filter out PD-L1-positive immune cells, which would otherwise be misdiagnosed as PD-L1-positive tumor cells. After optimization, the performance of the M2 model was greatly improved. As for the M3 workflow, the patches were first classified into four groups—tumor-positive patch, tumor-negative patch, immune positive patch, and other patch—followed by the detection of PD-L1-positive and PD-L1-negative cells using the YOLO model and then TPS calculation, as shown in **Figure 3**.

### Evaluation Metrics and Statistical Analyses

The linear correlation coefficient (LCC) was used for comparison with the TPS of AI models and the TPS given by the pathologists. Cohen’s kappa was also calculated for the agreement between pathologists and AI models. The Kappa values were interpreted as poor (<0.40), moderate (0.40–0.75), or excellent (≥0.75). Statistical significance was set at *p <*0.05. The accuracy evaluation was represented by several metrics, including specificity, sensitivity, precision, accuracy, and F1 score. All statistical analyses were performed using Python (version 3.6).

## Results

### Clinicopathological Characteristics of Patients With Lung Cancer

More than half of the sections (57.1%) were from male patients, and 42.9% were from female patients (**Table 1**). The sampling methods mainly included surgical operation, needle biopsy, other biopsy, and pleural effusion, and the total number of the corresponding samples was 617, 466, 141, and 31, respectively (**Table 1**). As for the tumor tissue types, there were 989 with lung adenocarcinoma and 185 with lung squamous cell carcinoma. The PD-L1 TPS (<1, 1–49, and ≥50%) and combined positive score (CPS) (<1 and ≥1%) assessments are also recorded in **Table 1**.

### DL Model Performance Evaluation in the PD-L1 (22C3) and PD-L1 (SP263) Assays

**Figure 4:**
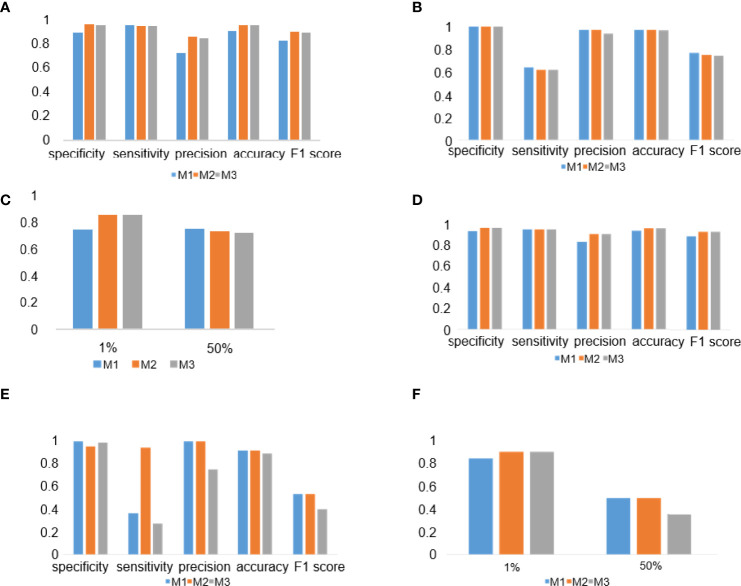
Deep learning (DL) model performance evaluation in the PD-L1 (22C3) and PD-L1 (SP263) assays. **(A–C)** Histograms of DL model performance with PD-L1 (22C3) assay test. **(D–F)** Histograms of DL model performance with PD-L1 (SP263) assay test. Tumor proportion score cutoff values of 1% **(A**, **D)** and 50% **(B**, **E)**. Kappa score analysis **(C**, **F)**.

To evaluate the performance of the experimental DL models, the test dataset was used for further analysis. TPS cutoff values of 1% (**Figures 4A, D**) and 50% (**Figures 4B, E**) were selected for the PD-L1 (22C3) (**Figures 4A–C**) and PD-L1 (SP263) (**Figures 4D–F**) assays in M1, M2, and M3. In the PD-L1 (22C3) assay, M2 and M3 showed improved performance in TPS calculation (**Figures 4A–C**), especially at 1% cutoff (M2: specificity, 0.9502; sensitivity, 0.9407; precision, 0.8523; accuracy, 0.9480; F1-score, 0.8944; kappa score, 0.8600; M3: specificity, 0.9457; sensitivity, 0.9407; precision, 0.8411; accuracy, 0.9445; F1-score, 0.8881; kappa score, 0.8510). Highly accurate performance in the PD-L1 (SP263) assay was also achieved for both M2 and M3 (M2: specificity, 0.9677; sensitivity, 0.9524; precision, 0.9091; accuracy, 0.9639; F1-score, 0.9302; kappa score, 0.9060 at 1% TPS cutoff values; M3: specificity, 0.9677; sensitivity, 0.9523; precision, 0.9091; accuracy, 0.9639; F1-score, 0.9302; kappa score, 0.9060 at 1% TPS cutoff values) (**Figures 4D–F**). Above all, the experimental DL models shown here obtained a high-precision score of PD-L1 expression.

### LCC in the 22C3 and SP263 Assays

To evaluate the consistency between the results of the DL model and the judgment of the pathologist, LCC was used for the analysis of M1, M2, and M3 in the PD-L1 (22C3) and PD-L1 (SP263) assays (**Table 2**). In the PD-L1 (22C3) dataset, M2 and M3 obtained a higher LCC score compared with M1 (M1: 95% CI, 0.791–0.844; M2: 95% CI, 0.858–0.892; M3: 95% CI, 0.854–0.892). A similar trend of the LCC score was also shown in the PD-L1 (SP263) set. The LCC values in M1, M2, and M3 of the PD-L1 (SP263) dataset were 0.825 (95% CI, 0.749–0.879), 0.867 (95% CI, 0.812–0.907) and 0.832 (95% CI, 0.766–0.882) respectively.

**Table 2:** Linear correlation coefficient in 22C3 and SP263 assay.

	Model	LCC	95% CI
22C3	M1	0.819	0.791–0.844
	M2	0.878	0.858–0.892
	M3	0.874	0.854–0.892
SP263	M1	0.825	0.749–0.879
	M2	0.867	0.812–0.907
	M3	0.832	0.766–0.882

Linear correlation coefficient among different artificial intelligence diagnostic models (M1, M2, and M3) in both PD-L1 (22C3) and PD-L1 (SP263) assays.

### Examples of Tumor Detection and PD-L1 Calculation

**Figure 5:**
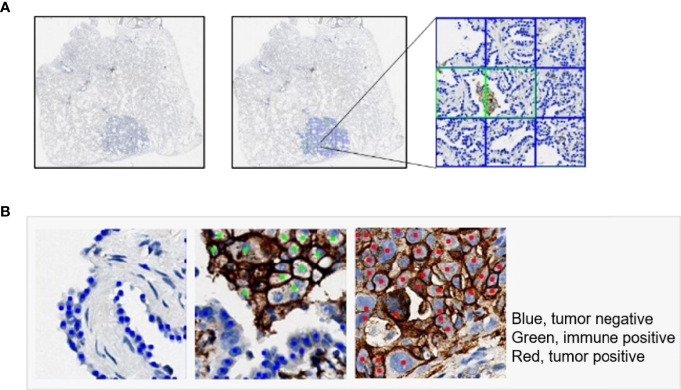
Examples of tumor detection and PD-L1 calculation. **(A)** Example of whole-slide image analysis for tumor recognition with the 256 patch. **(B)** Examples of cells detected by the YOLO model: PD-L1-negative tumor cell (blue), PD-L1-positive immune cell (green), and PD-L1-positive tumor cell (red).

An illustrative example of the process of tumor recognition with the 256 patch size is shown in **Figure 5A**. After obtaining PD-L1 IHC WSIs, tumor sections were detected and calculated by the DL model (**Figure 5A**). Moreover, based on pathological characteristics and PD-L1 staining, cells in the patches were further detected by object detection model and labeled with different colors for visualization (**Figure 5B**). Blue, green, and red represented PD-L1-negative tumor cells, PD-L1-positive immune cells, and PD-L1-positive tumor cells, respectively.

### PD-L1-Positive Immune Cell Patch Filter Module

**Figure 6:**
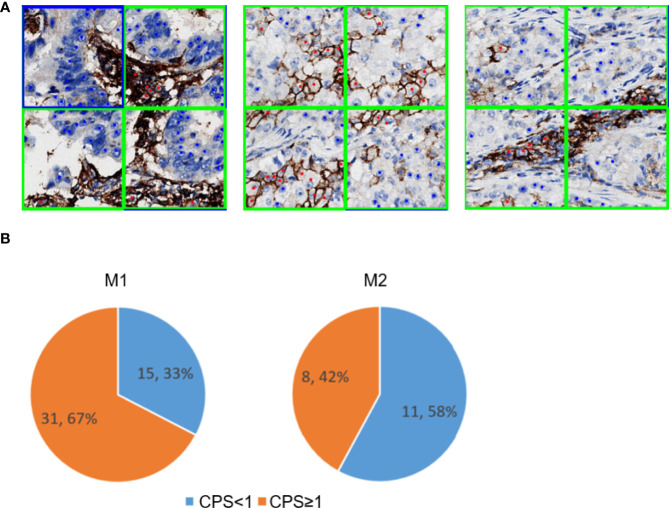
PD-L1-positive immune cells patch filter module. **(A)** Predicted tumor and immune patch annotated with blue and green squares, respectively. The predicted PD-L1-negative tumor cells and PD-L1-positive tumor cells are indicated with blue and red dots, respectively. **(B)** Performance of the immune filter module in M1 (left) and M2 (right). These pie charts show the percentage of false positive slides with CPS <1 and ≥1.

The effectiveness of the PD-L1-positive immune cell patch filter module in the M2 workflow is shown in **Figure 6**. The predicted tumor patch and immune patch are indicated with blue and green squares, respectively, and the predicted PD-L1-negative tumor cells and PD-L1-positive tumor cells are indicated with blue and red dots, respectively (**Figure 6A**). In the M1 workflow, most of the false positive samples of TPS have CPS ≥1% (**Figure 6B**). Compared with the M2 workflow, the M1 workflow does not have an immune filter module, so it usually leads to an increase in TPS because the M1 model easily misjudges PD-L1-positive immune cells as PD-L1-positive tumor cells (**Figure 6A**), which leads to a higher positive tumor ratio than normal (**Figure 6B**). The M2 workflow can greatly reduce the misjudging of PD-L1-positive immune cells as PD-L1-positive tumor cells through the filter module. Among the false positive samples in the M2 workflow, 42% of the samples have CPS ≥1 compared to that in the M1 workflow at 67%.

### DL Model Performance in Different Tumor Types and Surgical Methods

**Figure 7:**
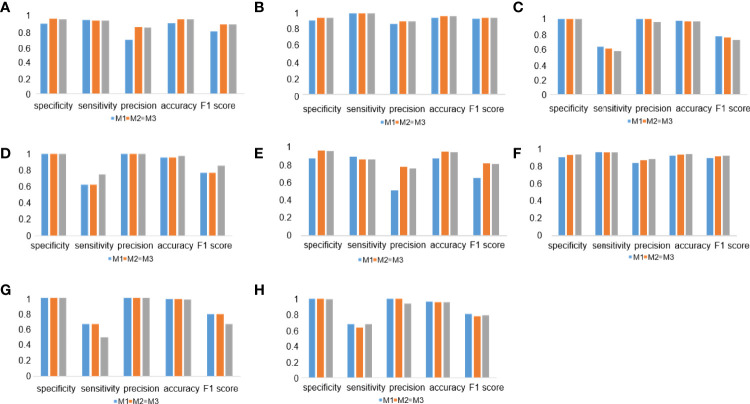
Deep learning (DL) model performance in different tumor types and surgical methods. **(A–D)** Histograms of DL model performance of lung adenocarcinoma **(A, C)** and lung squamous cell carcinoma **(B, D)** at the cutoff of 1% **(A, B)** and 50% **(C, D)**. **(D–F)** Histograms of DL model performance of samples from surgery **(E, G)** and needle biopsy **(F, H)** at the cutoff 1% **(E, F)** and 50% **(G, H)**.

To check the performance of the model more comprehensively, we stratified the testing results of the three workflows by different tumor types (**Figures 7A–D**) and surgical methods (**Figures 7E–H**). Compared with the M1 workflow, M2 and M3 showed better performance with TPS cutoff values of 1% in both lung adenocarcinoma (**Figure 7A**) (M2: specificity, 0.9569; sensitivity, 0.9320; precision, 0.8496; accuracy, 0.9517; F1-score, 0.8889; M3: specificity, 0.9543; sensitivity, 0.9320; precision, 0.8421; accuracy, 0.9497; F1-score, 0.8848) and lung squamous cell carcinoma (**Figure 7B**) (M2: specificity, 0.9024; sensitivity, 0.9615; precision, 0.8621; accuracy, 0.9254; F1-score, 0.9091; M3: specificity, 0.9024; sensitivity, 0.9615; precision, 0.8621; accuracy, 0.9254; F1-score, 0.9091). In terms of TPS cutoff values of 50%, M2 showed higher sensitivity but lower specificity when compared with M1 and M3 in both lung adenocarcinoma (**Figure 7C**) and lung squamous cell carcinoma (**Figure 7D**). A similar performance of M1, M2, and M3 in the 22C3 dataset was also shown in the samples from surgery (**Figures 7E, G**) and needle biopsy (**Figures 7F, H**). Thus, our DL models achieved a high-precision score of PD-L1 (22C3) in lung adenocarcinoma and lung squamous cell carcinoma in both sampling methods (surgery and needle biopsy).

## Discussion

An increasing number of studies have confirmed that PD-L1 is a critical predictive biomarker of non-small cell lung cancer (NSCLC) response to immunotherapy (29, 30). A higher percentage of positive PD-L1 expression is associated with a higher probability of responding to immunotherapy (31, 32). Thus, efficient and accurate assessment of PD-L1 expression is a critical step for clinical intervention. However, there are some problems with the traditional methods of evaluating PD-L1 expression levels. In this study, we developed three AI-based workflows that can be used for the quantitative scoring of PD-L1 expression in digital whole slides of lung cancer. These three fully automated AI-based workflows showed high specificity and accuracy in the PD-L1 expression of tumor cells, especially at 1% cutoff. Moreover, the high performance of our AI diagnostic models was further confirmed in lung adenocarcinoma and lung squamous cell carcinoma of both sampling methods (surgical sampling and needle biopsy).

In clinical practice, the experience of clinical pathologists has a certain impact on the identification of PD-L1 expression results (22). The assessment results of trained pathologists’ evaluation of PD-L1 expression are usually more accurate than those of untrained pathologists (33). It takes extensive professional learning and training for an untrained pathologist to become experienced (34). Previous studies have indicated that the assessment of PD-L1 expression by untrained pathologists has lower intraclass consistency compared with that by highly trained pathologists (35). Moreover, manually counting tumor cells for the interpretation of PD-L1 expression levels is of low efficiency and poor repeatability (24). Because of the intratumoral heterogeneity and variability of the whole slides of tumor tissues, it is a difficult task for a pathologist to get a precise assessment of all PD-L1-expressing tumor cells (36). Thus, the exploration of accurate and efficient automated diagnosis technology is urgently needed for the precise evaluation of PD-L1 expression in clinical practice.

The abovementioned challenges have been solved to a certain extent with the development of DL models (37). Previous studies have indicated that the diagnosis result of an AI model is highly consistent with that of highly trained pathologists and even better than that of untrained pathologists (22, 37). With fully automated labeling and calculation ability, a DL-based AI diagnostic model could assess the expression of PD-L1 (20) similar to a pathologist’s cognition of different cells. The exploration of the random forest assessment-based PD-L1 scoring algorithm indicated that the results of AI-based diagnostic models showed a high concordance with those of pathologists (38). Moreover, a previous study showed that different PD-L1-positive cells and other regions could be detected at a pixel level with a DL model (25). These results have indicated the promising application of the DL-based AI diagnostic model in PD-L1 scoring assessment for tumor immunotherapy in NSCLC.

However, the interpretation of slides with low PD-L1 expression is still a challenge for AI models (24, 25, 39). In this study, we focused on distinguishing PD-L1-positive tumor cells from PD-L1-positive immune cells. Our DL-based AI diagnostic workflows showed a high performance in PD-L1 scoring, especially at 1% cutoff. The M2 and M3 workflows showed high precision detection, and all five indicators performed well, especially for PD-L1 expression below 1%. We noticed that, in some cases, stromal cells in the tumor cell-containing patches were predicted as PD-L1-negative tumor cells in the YOLO model, leading to a lower TPS value and false negatives, especially at 50% cutoff value. In our proposed workflows, we compared two different patch sizes used in the classification models, namely, the 256 patch and the 128 patch. We speculated that the 128 patch would be superior to the 256 patch because cells in the 128 patch were more likely to be of the same type compared to the cells in the 256 patch, and this would reduce the counts of misdiagnosed stromal cells in the tumor cell-containing patches. However, the results shown in the classification models with these two different patch sizes almost have the same performance.

The tumor segmentation model reported in the literature requires a large amount of labeling work because it needs to be done at the pixel level (40). The patch classification model used in our study was easier to obtain. Moreover, it seems that the patch classification model has a strong ability to classify tissues with different structural patterns (41). In our study, compared to the M1 workflow, the M2 workflow ensured a higher accuracy of the results owing to its ability to distinguish PD-L1-positive tumor cells from PD-L1-positive immune cells. It is important to test the performance of AI models in samples with different histological subtypes or from different sampling methods encountered by pathologists in clinical practice. The AI diagnostic models shown here were tested in lung squamous cell carcinoma and lung adenocarcinoma, and the results show that there were no differences between histological subtypes. We also found that the proposed AI diagnostic models performed well both in samples from surgery and needle biopsy. Of the 18 pleural effusions samples, four negative samples were predicted as false positive, and one positive sample was predicted as false negative. Compared to the histological samples, the cytological samples have less structure information. It would pose a challenge for the AI model to distinguish tumor cells from other tissue cells in pleural effusions. In the future, more pleural effusion samples are needed to train our model and test its interpretation power of PD-L1 expression.

In current clinical practice, several assays for detecting PD-L1 expression using IHC analysis have been developed for different platforms, and some studies have evaluated various IHC assays for their reproducibility and sensitivity based on the respective scoring criteria (33). The results showed highly comparable staining by the 22C3, 28–8, and SP263 assays and lower sensitivity of the SP142 assay for determining TPS on TCs (33, 42). In this study, although the AI models were only trained with 22C3 immunostaining samples, our AI diagnostic models could effectively identify PD-L1-positive cells in both 22C3 and SP263 staining samples. In the future, it is valuable for us to evaluate the ability of the proposed AI diagnostic models in samples stained by other PD-L1 IHC assays.

There were some limitations in the current study. First, the sample size of this study was small, especially for pleural effusions. More slides need to be considered in a future study. Second, this study lacked multicenter external verification. Multicenter research could make our research results more convincing and could test the generalization ability of our AI models on slides from different sources. Third, the detection results of PD-L1 high-expression samples need to be further optimized. Fourth, factors such as the morphology and structural similarity between some PD-L1-positive immune cells and PD-L1-positive tumor cells as well as the presence of other confounding factors (*e*.*g*., poor quality of staining or the destruction of the structure of a tissue during sample preparation) may have affected the identification. It is still a challenge for the AI model to classify them. Immunostaining with more specific biomarkers (like CK for tumor cell or CD68 for macrophages) is a potential solution.

What is more, many studies have shown that PD-L1 expression alone is insufficient for patient selection in most malignancies, and a lot of new potential biomarkers are being studied for precision cancer immunotherapy (43). Increasing studies have indicated that the TME plays an important role in immunopathology and predicting clinical outcomes (44). Some predictive biomarkers in the components of TME have been widely used in immune checkpoint inhibitor therapies (45). The interactions between tumor and immune cells in the TME and their impact on the efficacy of tumor immunotherapy need more exploration. With the development of spatial TME profiling technologies (44), more comprehensive immunotherapy biomarker expression information will be further enhanced. DL is another emerging potential method that could assist in exploring the complexity of the TME, especially the spatial organization of tumor-infiltrating immune cells in the TME (14).

In summary, we explored and optimized three different AI model-based workflows for automatically detecting the positive PD-L1 expression in both 22C3 and SP263 assays. A highly accurate performance of the AI-assisted DL diagnostic models was shown in lung adenocarcinoma and lung squamous cell carcinoma of both sampling methods, especially for PD-L1 expression at 1% cutoff. Moreover, M2 could further improve the accuracy of the results. Our results indicate that AI-based diagnostic models are a promising approach to assist pathologists in making an accurate assessment of PD-L1 expression.

## Data Availability Statement

The original contributions presented in the study are included in the article/**Supplementary Material**, further inquiries can be directed to the corresponding authors.

## Ethics Statement

The studies involving human participants were reviewed and approved by The Ethics Committee of Zhejiang Cancer Hospital (IRB-2020-310 and IRB-2021-439). The patients/participants provided their written informed consent to participate in this study. The animal study was reviewed and approved by The Ethics Committee of Zhejiang Cancer Hospital. Written informed consent was obtained from the owners for the participation of their animals in this study. Written informed consent was obtained from the individual(s) for the publication of any potentially identifiable images or data included in this article.

## Author Contributions

Conceptualization: MW, QX, GC, PC, and DZ. Methodology and investigation: GC, FZ, YX, XH, HZ, SC, ML, PC, GD, and PC. Visualization: GC, PC, FZ, and YX. Funding acquisition: GC and MW. Project administration and supervision: MW, QX, GC, PC, DZ, and FZ. Writing—original draft: FZ, GC, and YX. Writing—review and editing: MW, QX, PC, and DZ. All authors contributed to the article and approved the submitted version.

## Funding

This work was supported by the Medical Health Science and Technology Project of Zhejiang Provincial Health Commission (2021KY579) and Zhejiang Provincial Natural Science Foundation of China under Grant (LGF20H160008).

## Conflict of Interest

FZ, YX, SC, ML, PC, DZ and CP are current or former employees of the company 3D Medicines Inc.

The remaining authors declare that the research was conducted in the absence of any commercial or financial relationships that could be construed as a potential conflict of interest.

## Publisher’s Note

All claims expressed in this article are solely those of the authors and do not necessarily represent those of their affiliated organizations, or those of the publisher, the editors and the reviewers. Any product that may be evaluated in this article, or claim that may be made by its manufacturer, is not guaranteed or endorsed by the publisher.

## Abbreviations

PD-L1, programmed cell death ligand 1; IHC, immunohistochemistry; DL, deep learning; AI, artificial intelligence; TME, tumor microenvironment; EGFR, epidermal growth factor receptor; HER2, human epidermal growth factor receptor 2; TPS, tumor proportion score; CNNs, convolutional neural networks; WSI, whole-slide image; LCC, linear correlation coefficient; CPS, combined positive score; IP, PD-L1-positive immune cells patch; NSCLC, non-small cell lung cancer; CK, cytokeratin.
