# Supplementary material for: Artificial Intelligence-Assisted Score Analysis for Predicting the Expression of the Immunotherapy Biomarker PD-L1 in Lung Cancer
Source: Front Immunol. 2022 Jul 1;13:893198. doi: 10.3389/fimmu.2022.893198 (PMC9286729; doi:10.3389/fimmu.2022.893198)
Supplement: Supplementary file 1 [file DataSheet_1.docx]

**Supplementary Figures**


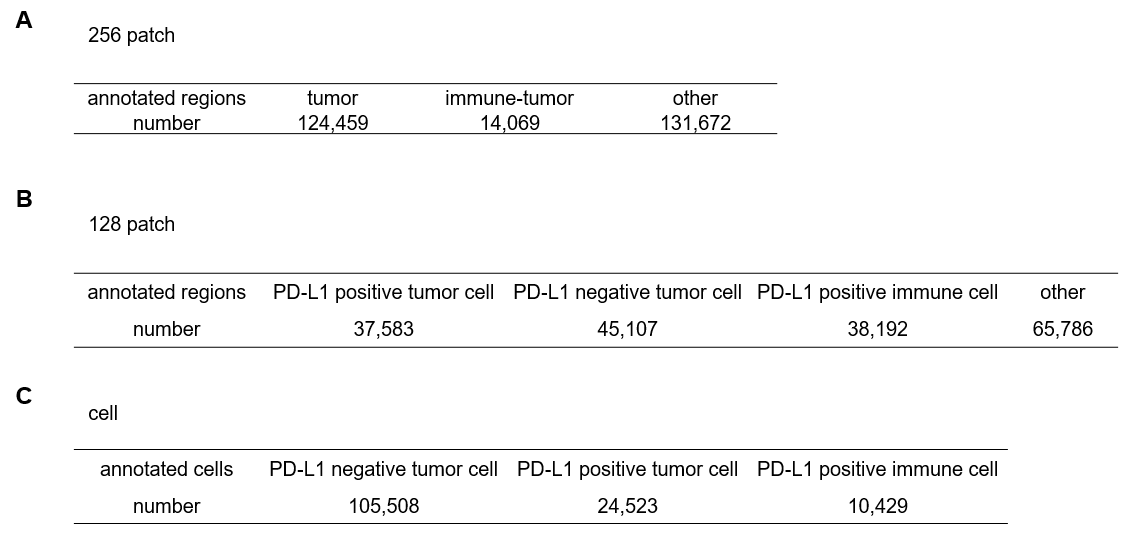


**Supplementary Figure 1. Training data set of different patches.**

(**A**) Typical regions annotated in the 256 patch. **(B)** Typical regions annotated in the 128 patch. **(C)** Cells annotated in the 256 patch.
